# Case Report: Idiopathic myointimal hyperplasia of mesenteric veins mimicking inflammatory bowel disease: a case report with literature review

**DOI:** 10.3389/fmed.2025.1674469

**Published:** 2025-11-10

**Authors:** Wei Wang, Xue Deng, Xin Jiang, Mengxue Yang, Xuefeng Tang

**Affiliations:** 1 Chongqing Medical University, Chongqing, China; 2 Department of Pathology, The Chongqing General Hospital, Chongqing, China

**Keywords:** idiopathic myointimal hyperplasia of mesenteric veins, *Clostridium difficile* infection, inflammatory bowel disease, idiopathic mesenteric phlebosclerosis, vessels with arterial-type wall thickening of uncertain significance

## Abstract

**Background:**

Idiopathic myointimal hyperplasia of the mesenteric veins (IMHMV) is a rare and poorly understood disease, typically affecting the rectosigmoid colon of young, otherwise healthy men. Clinically, it is often mistaken for inflammatory bowel disease, as biopsies show ischemic mucosal changes without classic inflammatory features. Surgical resection is both diagnostic and curative, although the etiology of IMHMV remains unclear.

**Case presentation:**

We report the case of a female patient with IMHMV involving the right hemicolon, concomitant with *Clostridium difficile* infection. Her symptoms persisted despite targeted treatment for *C. difficile*. She subsequently underwent a laparoscopic right hemicolectomy, which revealed mesenteric vein occlusion due to myointimal hyperplasia, confirmed by elastin staining and desmin immunohistochemistry. Histopathological examination established the diagnosis of IMHMV. The patient recovered well postoperatively, with no recurrence observed during follow-up.

**Conclusion:**

This is the first documented case of IMHMV involving the right hemicolon and complicated by *Clostridium difficile* infection. In addition, we reviewed 82 previously reported cases from 1991 to 2024, highlighting the clinical, imaging, and pathological characteristics of IMHMV. Recognition of this rare entity is essential to avoid unnecessary pharmacotherapy, prevent misdiagnosis as inflammatory bowel disease, and facilitate timely surgical management.

## 1 Introduction

Idiopathic Myointimal Hyperplasia of the Mesenteric Veins (IMHMV) is a rare and poorly understood condition that presents a diagnostic challenge for clinicians and pathologists. It is classified as an ischemic bowel disease characterized by venous occlusion due to smooth muscle proliferation in the tunica intima of the mesenteric veins, without the presence of a thrombus (1). IMHMV is often misdiagnosed as inflammatory bowel disease (IBD) (Supplementary Table S1), as definitive diagnosis of bowel ischemia and venous thrombotic disease relies on pathological changes that are not distinguishable through preoperative radiological or clinical assessments. Consequently, a definitive diagnosis can only be achieved postoperatively, since biopsies are unable to differentiate ischemic abnormalities from those associated with known IBD manifestations (2). Since the first case was reported in the United States in 1991, approximately 81 cases of IMHMV have been documented in the literature, with the present case representing the 82nd reported instance. Majority of these cases ultimately end up with different degrees of bowel resection (3).

Idiopathic myointimal hyperplasia of the mesenteric veins mostly involves the thickening of small and medium-sized mesenteric veins with the hallmark manifestation of intimal smooth muscle proliferation resulting in luminal occlusion and mucosal ischemic changes (2). Pathologists may miss the diagnosis unless elastin stains are performed, as the affected veins can easily be mistaken for arteries. The etiology of IMHMV remains unclear. One hypothesis suggests that the disease may result from an arteriovenous fistula. Another Hypothesis proposes that IMHMV represents the end stage of ‘phlebitis,’ as lesions resembling IMHMV have been observed in cases of lymphocytic, granulomatous, and necrotizing phlebitis (4, 5).

We herein describe the case of a female patient with idiopathic myointimal hyperplasia of the mesenteric veins (IMHMV) at right colon with *Clostridium difficile* infection (CDI).

## 2 Case presentation

A 68-year-old female patient, with a history of chronic abdominal pain, was admitted to the hospital with chief complaint of severe abdominal pain for the past 2 months. The abdominal pain initially started in the left hypochondrium as a vague discomfort, but later migrated and localized as a persistent distending pain in the lower abdomen, accompanied by vomiting, bloody stools, and low-grade fever. There was history of loss of appetite. She has consumed only porridge in the past 2 months. There was no history of joint pain, skin lesions, chest pain, cough, or contact with contaminated water. One month prior, she was admitted with a diagnosis of hypertensive heart disease, grade 3 hypertension, abnormal coagulation function, ischemic enteropathy, and chronic atrophic gastritis. She has previously undergone resection of a lung abscess. She self-reports an allergy to penicillins and abdominal pain after taking aspirin. No significant family history.

The physical examination revealed tenderness in the upper middle abdomen, left flank, and lower abdomen, more prominent in the upper middle and lower abdomen. No rebound tenderness or palpable masses were noted. Normal bowel sounds. The following Laboratory findings were reported: CRP = 20.51 mg/L, Hg = 106 g/L, D-Dimer = 0.78 mg/L, VIII factor activity = 267.90%, TP = 63.7 g/L, ALB = 31.8 g/L, positive fecal occult blood test, and *Clostridium difficile* antigen test.

**Figure 1:**
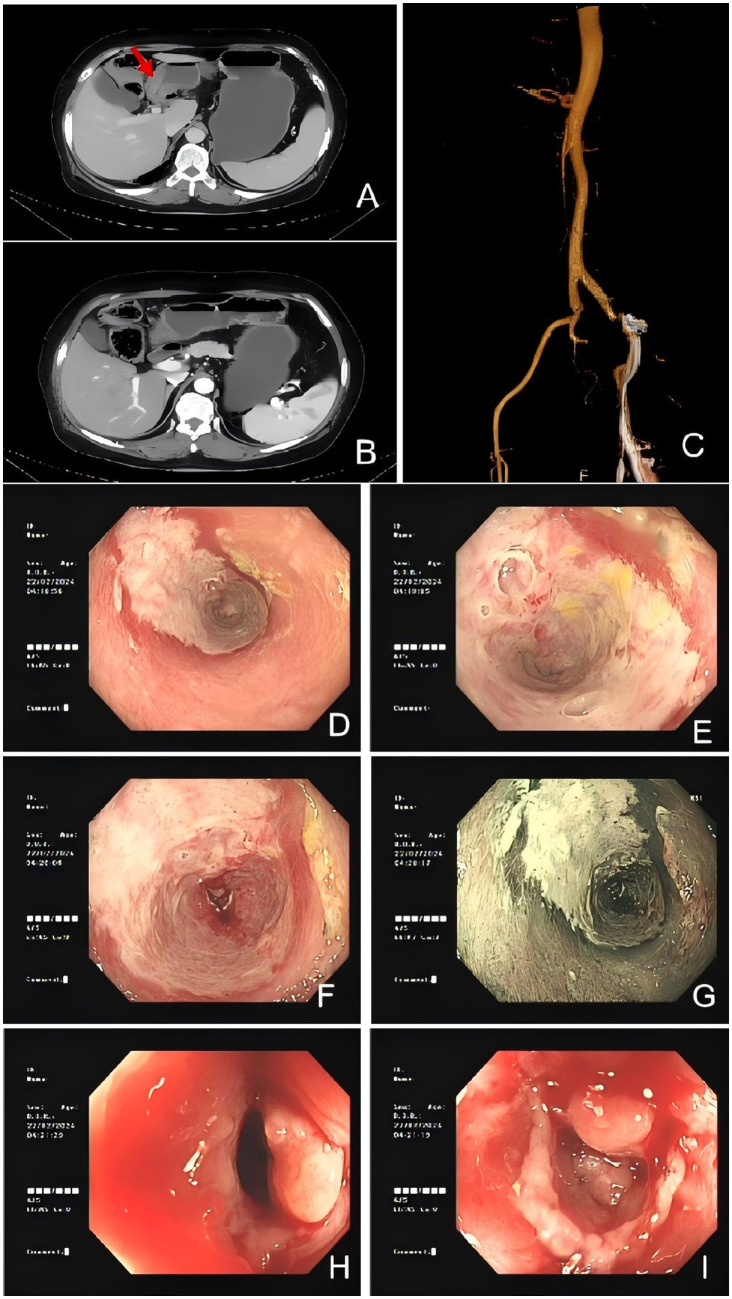
Colonic imaging and computed tomography suggested IMHMV. **(A,B)** Axial CT images showing thickened colonic wall with edema (red arrowhead) and no mesenteric artery stenosis. **(C)** Abdominal CTA revealed mixed plaques at the origin of the abdominal aorta and bilateral common iliac arteries, suggesting the possibility of mesenteric microthrombosis. Flexible sigmoidoscopy images demonstrated severely inflamed mucosa **(D–J)** with significant luminal narrowing **(H,I)**.

A colonoscopy revealed congested and inflamed mucosa in the colon (Figures 1D–I). Salt and pepper appearance was seen in the region. The transverse colon exhibited a whitish appearance with loss of vasculature. Biopsy findings revealed a non-specific ulcer.

Computed tomography angiography (CTA) of the abdomen showed mixed plaques in the abdominal aorta and at the origin of the bilateral common iliac arteries (Figure 1C). Computed tomography (CT) scan of the abdomen results showed a swollen colon wall with diminished enhancement and surrounding exudate that was suggestive of ischemic bowel disease (Figures 1A,B). Based on the presentation and investigative findings, she was diagnosed with a suspected case of ischemic bowel disease and was started on scopolamine hydrobromide for spasm and pain relief, fasting, omeprazole for gastric protection, and atorvastatin calcium tablets to stabilize plaques. The patient’s abdominal pain slightly subsided, but intermittent abdominal pain persisted, primarily around the navel, with slight relief after activity or oxygen inhalation. The stools were yellow and pasty. Papaverine was additionally used to relieve spasm and improve intestinal blood supply, while mesalazine was administered to repair the intestinal mucosa. Metronidazole tablets were added to treat the infection, but the patient experienced nausea and acid reflux after administration. Additionally, no pseudomembranous enteritis was found in the colonoscopy, and there was no mucus or pseudomembranes in the stool. Metronidazole was discontinued after 1 week. A conservative management approach was followed, but the symptoms did not resolve. A decision was made to proceed with a laparoscopic right hemicolectomy.

**Figure 2:**
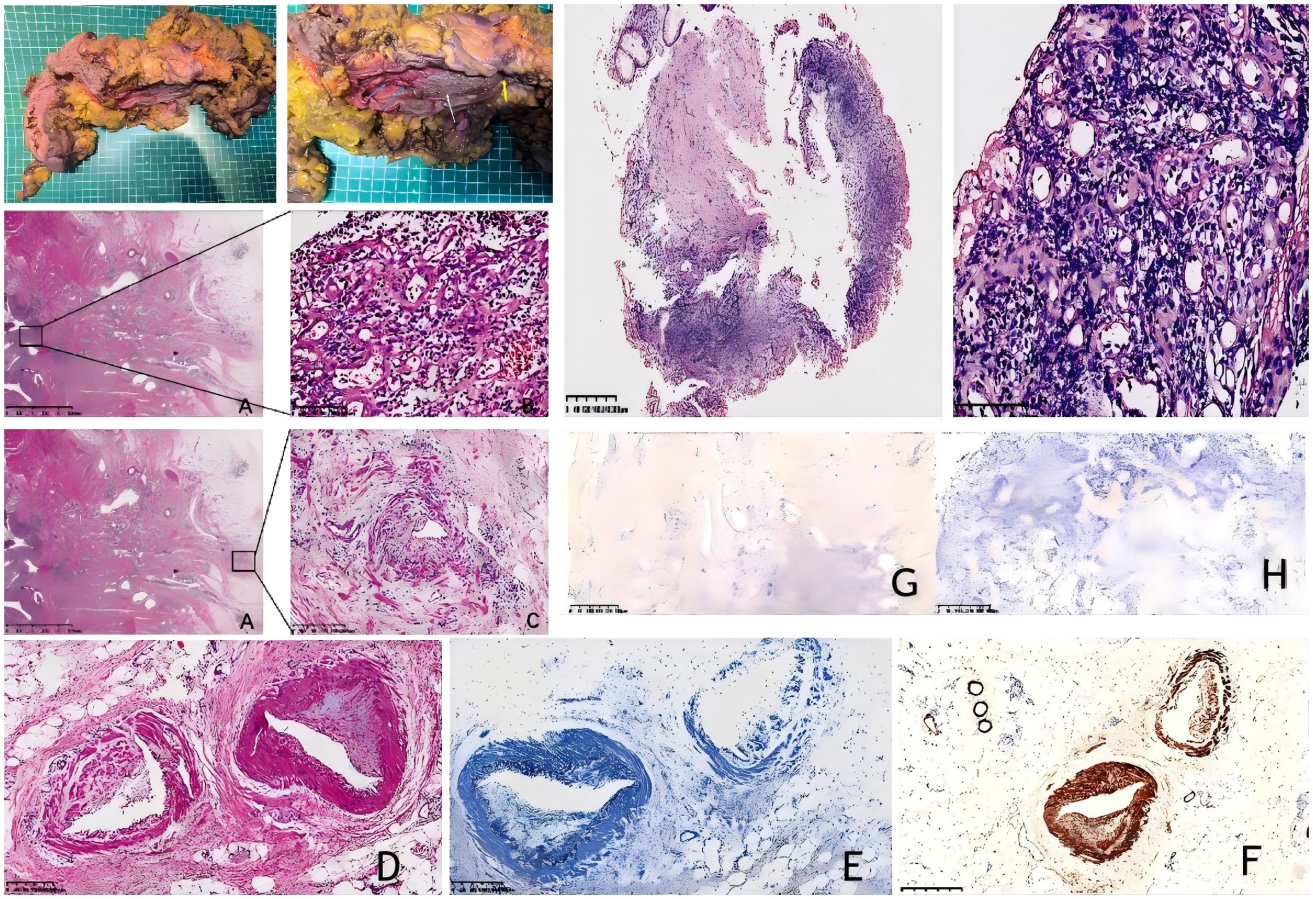
**(A–D)** Hematoxylin–eosin (H&E) staining of the resected surgical specimen (original magnification ×200); **(E)** elastic fiber staining (200x magnification); **(F)** immunostaining for smooth muscle actin shows the endoluminal nature of the venous proliferation. (200x magnification); **(G)** EBER *in situ* hybridization (ISH) test. (200x magnification); **(H)** CMV staining is negative. **(I&J)** Surgical resection specimen from IMHMV. Large ulcer (white arrow); scattered ulcers (blue arrow); stenosis (yellow arrow); **(K–M)** Hematoxylin–eosin (H&E) staining of the mucosal biopsy (original magnification ×200) shows ischemic-type changes, including epithelial sloughing, ulceration, and atrophy, with dense neutrophilic infiltration and prominent capillary proliferation but without perivascular hyalinization. The muscularis mucosae is thickened with associated fibrosis.

Postoperative pathology revealed the removal of 1 cm of ileum and 48 cm of colon. A local intestinal stricture was present 41 cm from the ileal resection margin, with a length of approximately 2 cm. The intestinal mucosa at this site was grayish-brown with granular hyperplasia. An ulcer was observed 28 cm from the ileocecal valve, adjacent to the stricture, measuring 10 × 3 cm. At this site, the intestinal mucosal folds were absent and the intestinal wall was hardened. The remaining proximal colon exhibited multiple scattered ulcers with a maximum diameter of 1–3 cm. The mesenteric fat at the lesion site was hyperplastic and encircled the intestinal wall, with the stricture being most prominent. Three lymph nodes were found around the intestine, with a maximum diameter of 0.2–0.3 cm (Figure 2).

### 2.1 Integrated histopathology description

Histopathological examination revealed that the intestinal mucosa and submucosa were replaced by fibrous tissue, with prominent proliferation of small- to medium-sized veins, some extending from the lamina propria to the serosa. The mucosal villi were absent at the stenotic segments, while the crypt architecture was largely preserved, without significant distortion or branching. Inflammatory infiltrates were present but limited, predominantly composed of neutrophils, with minimal lymphoplasmacytic component. Notably, there was no basal plasmacytosis, crypt abscesses, or Paneth cell metaplasia, features that typically indicate chronic inflammatory bowel disease, supporting the exclusion of IBD. The hyperplastic veins showed extensive intimal proliferation, resulting in slit-like lumina, with thickened walls and protruding endothelial cells. Capillaries in the affected areas also exhibited slit-like lumina and thickened walls. No histopathological features characteristic of *Clostridium difficile* infection, such as pseudomembranes, mucosal necrosis, or prominent exudates, were observed.

Immunohistochemical staining with Desmin confirmed that the smooth muscle in the venous intima was proliferating in a semicircular pattern. Concurrently, Verhoeff-Van Gieson (VVG) elastic fiber staining demonstrated noticeable intimal thickening in the arteriole-type vessels. No vasculitis was present; EBER RNA and CMV expression were negative, excluding EBV colitis and CMV colitis (Figure 2). No evidence of thrombosis or malignancy was observed. The final diagnosis was idiopathic myointimal hyperplasia of the mesenteric veins (IMHMV). Follow-up over 1 year after surgery, she was doing well, without issue.

We reviewed the patient’s previous biopsy specimens and noted dense neutrophilic infiltration of the mucosal layer with prominent capillary proliferation but without perivascular hyalinization. The muscularis mucosae was thickened with associated fibrosis and lacked features of chronic active inflammation, such as lymphoplasmacytic infiltrates, basement membrane disruption, or crypt abscesses. Although these findings were not diagnostic, they did not support inflammatory bowel disease or *Clostridium difficile* infection (Figures 2K–M).

## 3 Discussion

Idiopathic myointimal hyperplasia is a rare cause of intestinal ischemia. Compared to other inflammatory conditions of the gastrointestinal tract, IMHMV is not caused by arterial thromboembolism, venous thrombus or vasculitis, and its etiology remains poorly understood.

The most common symptoms reported in previous cases include abdominal pain, perforation, and hematochezia (Supplementary Table S1). The patient was admitted with chief complaint of severe abdominal pain. Patients with *C. difficile* enteritis generally present with diarrhea, crampy abdominal pain, and leukocytosis (6). Given that the patient presented with *Clostridium difficile* infection (CDI) and the clinical manifestations of IMHMV and pseudomembranous colitis caused by CDI were similar, the histological findings were distinctly different. The former often results in mucosal changes due to non-specific ischemic injury, rather than a toxin-mediated inflammatory process, with the absence of pseudomembrane formation and interstitial necrosis (7). CDI may lead to microvascular dysfunction and thrombosis of the superior mesenteric artery (8, 9) and affect the development of ischemic bowel disease (10). Ischemic colitis may be a complication of CDI (11). As a rare cause of ischemic bowel disease, CDI may offer insight into the pathogenesis of IMHMV. This case is notable as the first reported instance of IMHMV combined with CDI. CT imaging of our patient revealed focal colonic wall thickening and submucosal edema with preserved mesenteric arterial patency. However, the characteristic radiographic findings of IMHMV described in previous reports—enlarged and tortuous pericolonic vessels with rich, dilated peripheral veins—were not observed in this case (Figure 1). This absence may reflect the early stage and limited extent of venous involvement in our patient, as well as differences in imaging protocol compared with prior studies (Table 1).

**Table 1:** Clinical characteristics of all reported cases of IMHMV to date.

	Authors, year	Age(y)/Sex	Affected site	Clinical impression	Indication for surgery	Time to surgery	Follow-up
1	Current case	68/F	Right hemicolon	IC		2 months	
2	López Morales et al. (29)	37/M	Rectum to terminal ileum	CD	Abdominal pain	7 months	Died
3	Shah et al. (30)	24/F	Rectum to descending colon	IC	Abdominal pairvperforation		
4–15	Kim et al. (28)	Mean 66 (range 58–77/11M and 1F)	Rectosigmoid (*n* = 9), rectum to descending colon (*n* = 2) ileum to transverse colon (*n* = 1)	IC (*n* = 4); UC (*n* = 1),non-specific colitis (*n* = 1), CMV colitis (*n* = 1), ldiopathic Phlebosclero colitis (*n* = 1), IMHMV (*n* = 3)		Mean 3 months (range 1–8 months)	Mean 29 months (range 2–125 months)
16	Wong et al. (31)	72/M	Sigmoid to descending colon	IC	Abdominal pain		
17	Xie and Xu (32)	21/F	Rectosigmoid	IBD	Massive hematochezia	20 days	2 yr
18	Ansari et al. (33)	63/M	Sigmoid to descending colon	Entamoeba histolytica infection	Abdominal pain	>2 months	5 yr
19	Fang et al. (34)	21/F	Rectosigmoid	IBD	Hematochezia and perforation	2 months	1 yr
20	Yamada et al. (1)	81/F	Terminal ileum	Adhesive intestinal obstruction	Bowel obstruction		32 mo
21	Almumtin et al. (35)	55/M	Rectum to distal transverse	IBD	Perforation	1 yr	
22	Wu et al. (36)	53/M	Rectum to descending colon	UC	Persisting symptoms	3 months	3 months
23	Martin et al. (37)	63/M	Sigmoid to descending colon	IC/IBD	Persisting symptoms	5 months	2 months
24	Chudy-Onwugaie et al. (38)	54/M	Transverse colon	CMV colitis	Persisting symptoms	4 months	
25–32	Anderson et al. (39)	Median 62.5 (range 22–75)/6M and 2F	Sigmoid (*n* = 8)	IBD (*n* = 3)			
33	Louie et al. (40)	57/M	Small bowel		Abdominal pain		
34	Gonai et al. (41)	68/M	Sigmoid to descending colon	Mesenteric panniculitis	Persisting symptoms		
35–44	Yantiss et al. (3)	Mean 68 (range 25–83/9M and 1F)	Sigmoid to descending colon (*n* = 7), descending colon (*n* = 1), sigmoid colon(n = 2)	IC/IBD (*n* = 1), IBD (*n* = 7); IC (*n* = 2)	Perforation (*n* = 5), obstruction and refractory colitic symptoms		
45	Song and Shroff. (2)	59/M	Sigmoid to ileum	CD	Persisting symptoms	30 yr	2 wk
46	Yang et al. (42)	44/M	Rectosigmoid	UC	Persisting symptoms	4 wk	
47	Patel et al. (43)	65/M	Sigmoid to descending colon		Perforation	1.5 Months	
48	Guadagno et al. (44)	59/F	Ileum	CD	Multiple ileal neuroendocrine tumors	6 months	3 months
49	Costa et al. (45)	47/M	Rectosigmoid	IC/IBD	Persistent symptoms	9 months	
50	Cauchois et al. (46)	48/M	Rectum	IBD		3 months	
51	Yun et al. (4)	64/M	Rectum to distal transverse	UC	Hematochezia	2 yr	6 months
52	Wangensteen et al. (47)	62/F	Rectosigmoid	UC	Persistent symptoms	2 months	1.5 yr
53	Abbott et al. (48)	58/M	Rectum to descending colon	IC/IBD	Persistent symptoms		
54	Sahara et al. (12)	76/M	Rectosigmoid	IC/IBD	Persistent symptoms	1 yr	3 months
55	Laskaratos et al. (49)	62/F	Ileum	IBD	Perforation and hematochezia		
56	Zijlstra et al. (50)	62/M	Rectum to descending colon		Acute abdomen		2 yr
57	Korenblit et al. (51)	59/M	Rectosigmoid	IC	Persistent symptoms	1 month	3 months
58	Feo et al. (52)	75/F	Rectosigmoid	IC	Persistent symptoms	6 months	
59	Lanitis et al. (53)	81/M	Terminal ileum		Appendiceal mucocoele and pseudomyxoma peritonei	6 months	
60	Korenblit et al. (51)	62/M	Entire colon (rectal sparing)	UC	Hematochezia	18 months	
61	Chiang et al. (54)	60/M	Rectosigmoid	UC	Persistent symptoms	2 months	4 months
62	Garciaos et al.	32/M	Rectum to descending colon	primary pneumatosis intestinalis	Abdominal pain and hematochezia	3 months	24 months
63	Kao at al. (55)	38/M	Rectosigmoid	IBD	Perforation	5 months	18 months
64	Savoie and Abrams (56)	22/M	Rectosigmoid	IBD	Abdominal pain and hematochezia		10 months
65	Bryant et al. (14)	42/F	Jejunum	/			
66	Abu-Alfa et al. (5)	58/M	Sigmoid	IC/IBD	Abdominal pain and hematochezia	1 yr	
67–70	Genta and Haggit et al. (57)	Mean 40 (range 25–67)/4 M	Sigmoid (*n* = 1), Sigmoid to descending colon (*n* = 1), Rectosigmoid (*n* = 2)	UC (*n* = 2), CD (*n* = 1); Stricture (*n* = 1)	Bowel obstruction (*n* = 1), toxic megacolon (*n* = 1),abdominal pain and hematochezia (*n* = 2)	Mean 3 months (range 1-6 months)	Mean 3.5 yr. range 1–7 yr
71	Li et al. (58)	64/M	Ileum	IBD	Bowel obstruction	6 months	1 yr
72	Shi et al. (59)	25/M	Small bowel	CD	Bowel obstruction	10 yr	5 months
73	Noujaim et al. (62)	66/M	Rectum to descending con	IBD	Persistent symptoms	1 month	Died
74	Morimura et al. (60)	44/M	Left hemicolon		Intestinal ischemia and necrosis	1 month	1 yr
75	Kawasaki et al. (27)	71/M	Rectosigmoid	IMHMV		4 months	3 months
76–79	ROBERT M et al.	Median 38 (range 38–67)/4 M	Rectosigmoid		Persistent symptoms	Mean 1 months (range 1 months-1 yr)	
80–81	Bhatt et al. (61)	82,59/2 M	Sigmoid to descending colon		Intestinal ischemia and necrosis	3 months	7 yr
82	Huanhuan Xie	21/F	Rectosigmoid		Persistent symptoms	2 weeks	1 yr

IMHMV, idiopathic myointimal hyperplasia of mesenteric veins; IBD, inflammatory bowel disease; M, male; F, female; yr, year.

Our patient has hypertension and hypertensive heart disease, consistent with previous reports noting cardiovascular risk factors in many IMHMV cases (4, 12–14). IMHMV is characterized by myointimal hyperplasia leading to mesenteric vein occlusion. Experimental studies have shown that elevated blood pressure can induce intimal thickening and endothelial changes in small vessels (15–26), which may suggest a potential role of hypertension in the pathogenesis of IMHMV. Further research is warranted to clarify this association.

The absence of concrete histopathological criteria for a definitive diagnosis of IMHMV makes biopsy-based diagnosis challenging for pathologists, leading to the early initiation of treatment with anti-inflammatory drugs rather than surgery, which, at present, remains the only effective treatment for this condition and has been reported to be completely curative. However, only six cases of IMHMV have been diagnosed preoperatively (27–29). Arteriolized capillary, subendothelial fibrin deposits, and perivascular hyalinization are the most recent specific pathological features that facilitate the identification of IMHMV in mucosal biopsy samples (1). Biopsy specimens from this patient were reviewed to summarize findings indicating the absence of the muscular layer in the intestinal wall, a gap-like appearance of the intestinal wall due to capillary hyperplasia, thickening of the wall, and prominent endothelial cells. Notably, while the mesenteric vein exhibited characteristic smooth muscle proliferation in the intima (consistent with IMHMV pathology), the arterial changes presented a diagnostic paradox: although these vessels retained their intimal architecture, significant non-myointimal thickening was observed, which differed fundamentally from the venous pathology. we describe these as vessels with arterial-type wall thickening of uncertain significance, confirmed by immunohistochemistry to be non-myointimal in nature (Figure 2). This histologic feature has not been documented in prior IMHMV cases and warrants particular attention, as it may represent (1) a previously unrecognized disease variant, (2) concurrent vascular pathology, or (3) a broader disease spectrum or secondary vascular remodeling, given that small-vessel involvement of this type has not been reported in the existing literature. The presence of definitive venous pathology meets the current diagnostic criteria for IMHMV, while the observed alterations in other small vessels may indicate secondary vascular remodeling, a coexisting process, or a previously unrecognized disease spectrum that requires further investigation.

Currently, all cases are diagnosed based on pathological results, and the lack of methods for early diagnosis means that the diagnosis of IMHMV is often delayed. The clinical manifestations and endoscopic findings are nonspecific and closely resemble those of IBD and IMP. Similar imaging manifestations have also been observed, with IMP showing more similarity in this regard. Below, we summarize the differential diagnosis of IMHMV, IBD and IMP (Table 2). In particular, IBD, with nearly 53.1% of IMHMV cases being diagnosed as IBD before surgery (Supplementary Table S2), will be subdivided into the two subtypes of IBD, namely UC and CD, to differentiate them from IMHMV. This approach can enhance clinicians’ understanding of the disease and improve the diagnostic rate.

**Table 2:** Differential diagnosis of IMHMV.

	IMHMV	UC	CD	IMP	MAD/AVD
Onset age	Older (mean age 58 years old)	Younger (20–40 years old)	Younger (18–35 years old)	Older (30–86 years old)	Younger (variable, often <50) Usually younger adults (variable, often <50)
Clinical features	Abdominal pain > hematochezia > diarrhea, complicated with intestinal bleeding and perforation	Hematochezia > diarrhea > abdominal pain	Diarrhea > abdominal pain > weight loss, perianal involvement and extraintestinal manifestations are common	Abdominal pain > nausea or vomiting > diarrhea, abdominal distension and constipation and hematochezia	Chronic abdominal pain, GI bleeding; may have ischemic symptoms
Sites of involvement	Rectosigmoid and descending colon, rarely in small intestine	Rectosigmoid, develops from distal to proximal colon	Terminal ileum and ileocecum > colon > rectum > small intestine > upper digestive tract	Right hemicolon, the ascending colon is most susceptible	Variable, can involve small intestine and colon
Skipped lesions	No	No	Yes	No	Yes
Ulcers	Non-specific ulcers	Superficial ulcers	Longitudinal ulcers, cobblestone appearance and aphthous ulcers	Non-specific ulcers	ischemic-type ulcers; may resemble Crohn’s disease
Histopathology	Intima and media smooth muscle proliferation	Crypt abscess, cryptitis, superficial ulcers, and plasma cells increase in the basal layer	Non-caseating granuloma	Mucosa fibrosis, hyalinoid degeneration, the venous wall fibrotic thickening, calcification,	Arteriovenous malformations: thickened arterial and venous walls, abnormal capillary connections; mixed arterial and venous involvement
Treatment	Surgery, no response to medication	Response to 5-Aminosalicylic acid, steroid, immunosuppressant or biologic agents	Response to 5-Aminosalicylic acid, steroid, immunosuppressant or biologic agents	stopping the use of herbs and symptomatic management	Variable; sometimes endovascular intervention or surgery needed
Recurrence post operation	No	Yes	Yes	No	Possible, depending on completeness of resection or persistence of vascular malformation

IMHMV, idiopathic myointimal hyperplasia of mesenteric veins; UC, Ulcerative colitis; CD, Crohn’s disease; IMP, idiopathic mesenteric phlebosclerosis; MAD/AVD, Mesenteric Arteriovenous Dysplasia/Vasculopathy.

## 4 Conclusion

This case report describes the first documented instance of Idiopathic Myointimal Hyperplasia of the Mesenteric Veins (IMHMV) with right colonic involvement complicated by *Clostridium difficile* infection in a female patient, and also provides the inaugural description of small vessels exhibiting arterial-type wall thickening of uncertain significance in IMHMV.

Histopathological analysis demonstrated a paradoxical coexistence of characteristic venous myointimal hyperplasia and a subset of small vessels showing arterial-type wall thickening that distinctly lacked Desmin-positive smooth muscle cell proliferation—an essential feature of the venous lesions—thus creating a diagnostic paradox given the established venocentric pathology of IMHMV. Clinicians should maintain a high index of suspicion for IMHMV when encountering venocentric myointimal hyperplasia in biopsy specimens from elderly patients with suspected inflammatory bowel disease or distal colorectal ischemia, with the caveat that concurrent arterial-type wall thickening of uncertain significance should prompt thorough clinicopathological correlation to exclude alternative vasculopathies.

## Data Availability

The original contributions presented in the study are included in the article/Supplementary material, further inquiries can be directed to the corresponding author/s.
