# Supplementary figures and images for: Case Report: Idiopathic myointimal hyperplasia of mesenteric veins mimicking inflammatory bowel disease: a case report with literature review
Source: Front Med (Lausanne). 2025 Nov 10;12:1674469. doi: 10.3389/fmed.2025.1674469 (PMC12640971; doi:10.3389/fmed.2025.1674469)

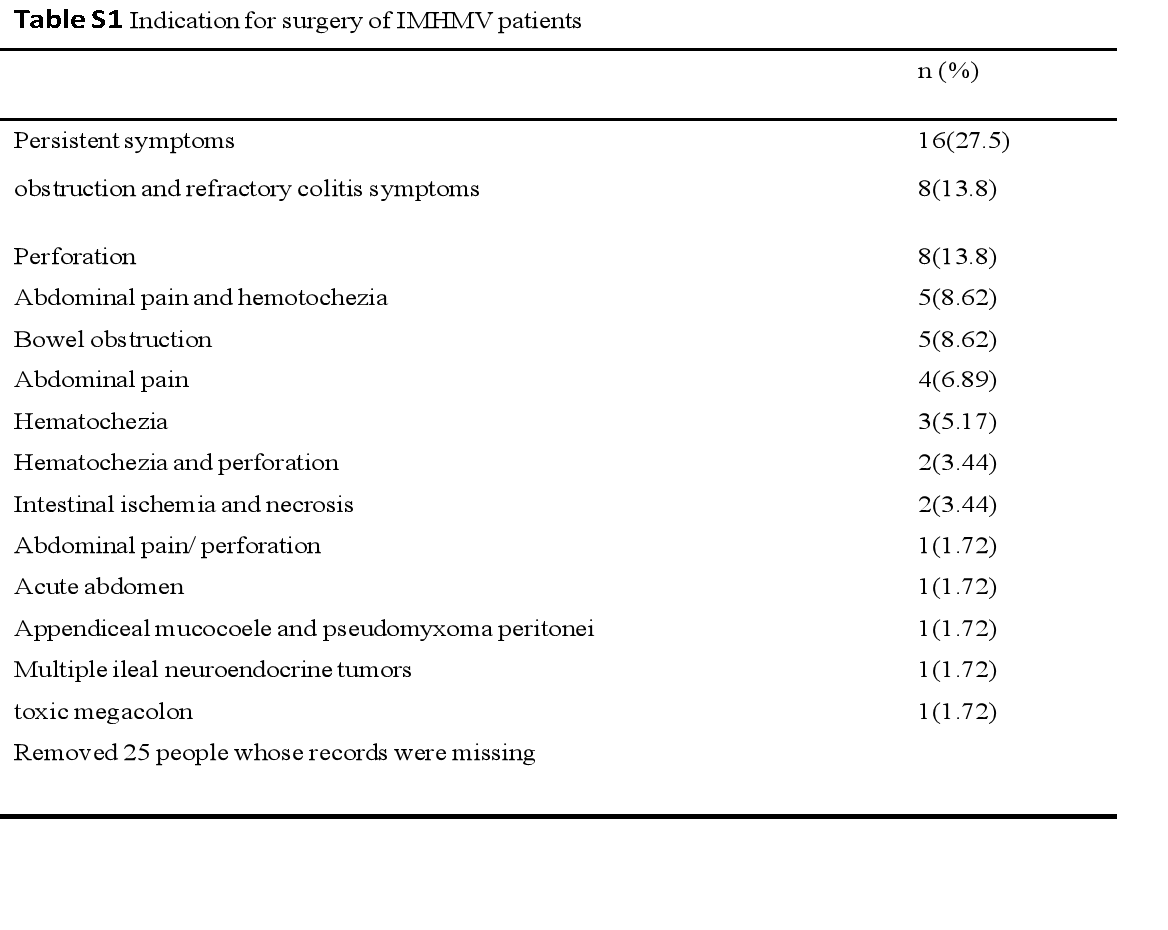

Supplement: Supplementary file 1 [file Image_1.TIF]

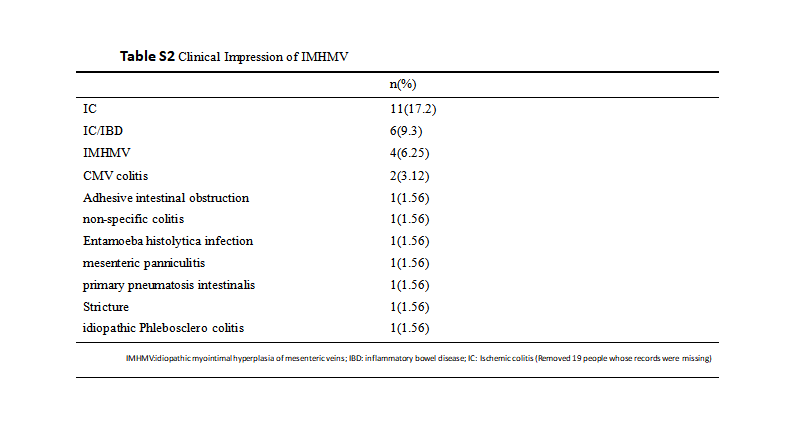

Supplement: Supplementary file 2 [file Image_2.TIF]
